# Analysis of risk factors for death during treatment of hemodynamically unstable pelvic fractures

**DOI:** 10.12669/pjms.41.2.9808

**Published:** 2025-02

**Authors:** Dazhi Wang, Jungang Xiao, Kaifu Zheng

**Affiliations:** 1 Dazhi Wang, Trauma Orthopedics, China Resource & WISCO General Hospital, 430080, Wuhan, China; 2 Jungang Xiao, Trauma Orthopedics, China Resource & WISCO General Hospital, 430080, Wuhan, China; 3 Kaifu Zheng, Trauma Orthopedics, China Resource & WISCO General Hospital, 430080, Wuhan, China

**Keywords:** Hemodynamic instability, Logistic, Pelvic fracture, Risk factor

## Abstract

**Objective:**

To analyze the risk factors associated with the occurrence of death during the treatment of patients with hemodynamically unstable pelvic fractures.

**Methods:**

The present study is a retrospective research design, which selected 136 patients with hemodynamically unstable pelvic fractures admitted to China Resource & WISCO General Hospital from March 2020 to March 2022 as the subjects of the study. They were divided into a survival group (113 cases) and a death group (23 cases) according to whether the patients died during treatment. The general clinical data and laboratory test indexes of the two groups were compared to analyze the risk factors affecting the death of pelvic fracture patients. Receiver operator characteristic (ROC) curves were plotted, and the area under the curve was calculated.

**Results:**

The two groups were significantly different in aspects of age, systolic blood pressure, rapid emergency medicine score, injury severity score (ISS), sequential organ failure assessment (SOFA) score, prothrombin time, activated partial thromboplastin time, and percentages of patients with bleeding volume > 2,000 mL, shock index > 2, and minimum oxygenation index < 200 (P<0.05). Logistic regression analysis suggested that shock index > 2, ISS, SOFA score, and bleeding volume > 2,000 mL were independent risk factors for patient death. ROC analysis showed that the area under the curve for shock index > 2, ISS, SOFA score, and bleeding volume > 2,000 mL was 0.656, 0.732, 0.907, and 0.798, respectively.

**Conclusions:**

Severe shock, increased ISS and SOFA score, and bleeding are independent risk factors for death in patients with hemodynamically unstable pelvic fractures. Implementation of rapid and efficient targeted management during treatment of high-risk patients with these factors is key to reducing their risk of death.

## INTRODUCTION

The pelvis is the support system of the spine and an important bony structure that connects the human trunk to the lower extremities.^1^ The pelvis is highly stable, and except for special cases such as pathologic fractures and osteoporosis, pelvic fractures are mostly caused by high-energy injuries such as motor vehicle accidents and falls, and are usually associated with combined multisystem injuries that can have catastrophic consequences.^2^,^3^ A study has reported that the high incidence of pelvic fractures with multiple injuries in recent years is mainly related to the rapidly developing transportation and construction industries in China.^4^

The percentage of unstable fractures caused by high-energy external factors in pelvic fractures is more than 65%, and patients with such kind of fracture are often associated with severe blood loss, organ damage, and other pathological damage caused by multiple injuries, resulting in hemodynamic instability.^5^ The lateral compression 3 (LC3) injury in the Young-Burgess classification, the anterior-posterior compression 2 (APC2) and APC3 injuries, the vertical compression (VS) injury, and the compound (CM) injury are all hemodynamically unstable fracture types. Among different Tile types, B3 and C fractures are hemodynamically unstable pelvic fractures.^6^ A study has shown that the mortality rate of hemodynamically unstable pelvic fractures is 30% to 50%.^7^ Therefore, it is important to quickly, accurately, and efficiently identify the injury in these patients at an early stage and provide timely and targeted management.

A study found that,^8^ in addition to blood loss, untimely resuscitation and inadequate resuscitation measures are important factors contributing to the death of patients with hemodynamically unstable pelvic fractures. However, there are fewer studies on the predictive factors of death in this category of patients in China and abroad, and the existing studies mostly analyze the therapeutic measures for pelvic fractures, lacking a profound understanding of the morbidity characteristics, treatment and prognosis of this category of patients.^9^

This study collected clinical data of 136 patients with hemodynamically unstable pelvic fractures in our hospital and analyzed the risk factors that may affect the death of these patients, to facilitate the clinical identification of patients with risk factors for death, with the aim of proposing measures to improve the pre-hospital emergency and in-hospital treatment of patients with hemodynamically unstable pelvic fractures and to improve the level of pelvic trauma treatment. This paper provides a reliable basis for further reducing mortality and disability rates, halting disease progression, and improving patient prognosis.

## METHODS

In this retrospective study, 136 patients with hemodynamically unstable pelvic fractures [Type-C according to the World Society of Emergency Surgery (WSES) classification, i.e. WSES Grade-IV] admitted to China Resource & WISCO General Hospital between March 2020 and March 2022 were selected.

### Inclusion criteria

were definite diagnosis of pelvic fracture, systolic blood pressure (SBP) < 90 mmHg, complete clinical data, informed consent for this study, conscious, and no communication impairment.

### Exclusion criteria

were pathological fracture, serious immune diseases, malignant tumors, and serious underlying diseases that could not tolerate surgery. There were 97 males and 39 females with a mean age of (49.02±15.82) years.

Patients who improved within 30 days of hospitalization were classified as the survival group, i.e., those who were in stable condition or were transferred to other departments or other hospitals for follow-up treatment or elective surgery. Patients who deteriorated and died within 30 days of hospitalization were classified as the mortality group, i.e., those who were not stable enough to be transferred out of the intensive care unit or who died during treatment despite active treatment within 30 days of hospitalization. There were 113 cases in the survival group and 23 cases in the death group.

### Ethical Approval:

This study was approved by the ethics committee of our hospital (No. HRWGZYY2023005J dated on July 10^th^ 2023).

### Observed indicators:

Preoperative general information: age, sex, heart rate, blood pressure, body temperature, shock index, minimum oxygenation index, cause of injury, Tile typing, and bleeding volume.

### Patient admission scores:

Injury severity score (ISS), sequential organ failure assessment (SOFA), and rapid emergency medicine score (REMS).

### Laboratory parameters:

White blood cells (WBC), hemoglobin (HBG), platelet (PLT) count, prothrombin time (PT), and activated partial thromboplastin time (APTT) within 24 hours of admission.

### Patient treatment:

Surgical method.

### Statistical analysis:

SPSS25.0 was used for statistical analysis of the data. Measurement data that conformed to a normal distribution were expressed as (*χ̅*±*S*) and compared using the two independent samples t-test; measurement data that did not conform to a normal distribution were expressed as M (P25, P75) and compared using the two independent samples rank sum test. Count data were expressed as percentages and compared using the X^2^ test or continuous correction X^2^ test. Factors with significant differences (P<0.05) in the univariate analysis were included in the logistic regression analysis to further identify independent risk factors affecting patient prognosis. ROC curves were plotted for the relationship between these factors and patient death. Differences were considered statistically significant at P<0.05.

## RESULTS

The factors related to the possible influence of death in patients with pelvic fractures were compared, and the results showed that there were statistically significant differences between the two groups in terms of age, SBP level, REMS, ISS, SOFA score, PT, APTT, surgical method, etc. (P<0.05); the surviving group had bleeding volume > 2,000 mL, shock index > 2, and lowest oxygenation index < (the percentage of patients with 200 was lower than that of the mortality group, and the difference was statistically significant P<0.05); the differences between the two groups in terms of gender, heart rate, blood pressure, body temperature, cause of injury, classification of pelvic fractures, white blood cells, hemoglobin and platelet count were not statistically significant (P>0.05) Table-I.

**Table-I:** Univariate analysis of factors affecting patient mortality.

Possible influencing factors		Survival group (n=113)	Death group (n=23)	t/X^2^	P
Age (years)		47.47±15.56	55.41±15.61	2.017	<0.05
Gender	Male	88 (77.88)	19 (82.61)	2.209	>0.05
	Female	25 (22.12)	4 (17.39)		
Heart rate < 60 beats/min		29 (25.67)	7 (30.43)	1.746	>0.05
Bleeding volume > 2,000 mL		14 (12.39)	21 (91.30)	16.715	<0.05
Body temperature > 38.5°C		30 (26.55)	8 (34.78)	2.489	>0.05
SBP (mmHg)		94.6 (81.6, 102.26)	83.2 (78.8, 89.23)	2.486	<0.05
Shock index > 2		15 (13.27)	15 (65.22)	15.631	<0.05
Minimum oxygenation index <200		14 (12.39)	11 (47.83)	13.548	<0.05
SOFA		6.6 (4.1, 10.1)	15.7 (12.2, 18.2)	5.267	<0.05
ISS		23 (23, 28)	29 (25, 35.5)	3.247	<0.05
REMS		12.09±5.16	24.74±6.36	8.304	<0.05
WBC (10^9^ /L)		11.85 (7.81, 15.62)	10.48 (6.21, 15.03)	1.195	>0.05
HBG (g/L)		85.61±19.52	84.59±15.10	0.713	>0.05
PLT (10^9^ /L)		90.5 (59.75, 141.75)	83.6 (33.74, 111.65)	1.487	>0.05
PT (s)		13.30 (12.18, 14.82)	17.5 (15.35, 18.05)	3.572	<0.05
APTT (s)		30.8 (27.27, 35.55)	38.17 (30.09, 58.8)	3.339	<0.05
Cause of injury	Traffic accidents	70 (61.95)	13 (56.52)	0.657	>0.05
	Falling	32 (28.32)	7 (30.43)		
	Hit by a heavy object	13 (11.50)	3 (13.04)		
Classification of pelvic fractures [n(%)]	Tile A	58 (51.33)	11 (47.83)	0.886	>0.05
	Tile B	37 (32.74)	8 (34.78)		
	Tile C	18 (15.93)	4 (17.39)		
Surgical treatment	Pelvic surgery is not performed	60 (53.10)	14 (60.87)	11.927	<0.05
	Internal pelvic fixation is performed	36 (31.86)	2 (8.70)		
	External pelvic fixation stent is performed	17 (15.04)	7 (30.43)		

Multifactorial logistic regression analysis showed that shock index > 2, ISS, SOFA score, and bleeding volume > 2,000 mL were all independent risk factors for death in patients with hemodynamically unstable pelvic fractures (Table-II).

**Table-II:** Multifactorial analysis related to patient death.

Influencing factor	OR	95% CI	P
Shock index > 2	1.171	1.026~1.338	0.019
ISS	1.308	1.003~1.705	0.046
SOFA score	1.889	1.053~3.387	0.032
Bleeding volume > 2,000 mL	1.028	1.004~1.055	0.029

**Fig.1:**
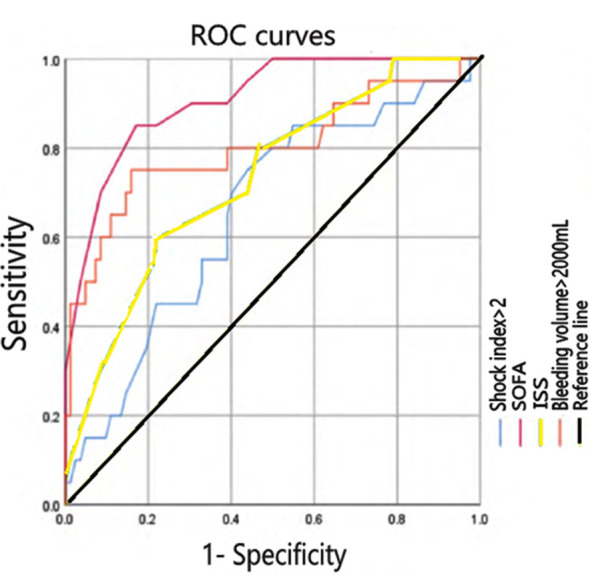
ROC curves of independent risk factors.

Receiver operator characteristic (ROC) curves were plotted for the relationship between independent risk factors and patient death. The area under the curve (AUC) for patient shock index, ISS score, SOFA score, and bleeding were 0.656, 0.732, 0.907, and 0.798, respectively; the sensitivity was 76%, 59%, 86%, and 76%, respectively; the specificity was 56.2%, 78.1%, 83.0%, and 84.2%, respectively (Fig.1, Table-III).

**Table-III:** ROC analysis with statistically significant continuous variables.

Influencing factor	AUC (95% CI)	Sensitivity	Specificity	P
Shock index > 2	0.656 (0.523-0.788)	76%	56.2%	0.032
ISS	0.732 (0.610-0.850)	59%	78.1%	0.001
SOFA score	0.907 (0.841-0.976)	86%	83.0%	<0.001
Bleeding volume > 2,000 mL	0.798 (0.587-0.918)	76%	84.2%	<0.001

## DISCUSSION

This study retrospectively analyzed the clinical data of patients with hemodynamically unstable pelvic fractures and found that the main age distribution of injured patients was 20-59 years, with a predominance of males, and traffic accident injuries and fall injuries were predominant, which was consistent with the findings reported by Yang et al.^10^ This study also found that shock index, ISS, SOFA score, and bleeding volume were independent risk factors for patient death. The shock index reflected the overall severity of the patient’s injury and was also the main factor contributing to the patient’s death, which was similar to the results of previous studies. The results of a study by Shi et al. showed that the higher the shock index,^11^ the more severe the injuries of patients with pelvic fractures and the more likely they were to die.

The ISS system assesses the severity of human trauma from six anatomical sites: head and neck, maxillofacial, thorax, abdomen and pelvis, extremities and pelvis, and body surface.^12^ It has been reported in the literature that the mortality rate of trauma patients increases as the ISS increases.^13^,^14^ In addition, another study found that the higher the ISS,^15^ the more likely the patient was to develop the death triad. The present study also showed that the higher the ISS, the higher the risk of death of the patients, but it did not mention the death triad. The general information of the study subjects in this study was less comprehensive than the above-mentioned studies. The above issues will be deeply studied in the future.

The SOFA score objectively assesses the functional status of different organs of the patient by scoring different items of the six major systems of the body (respiratory, hematological, hepatic, cardiovascular, neurological, and renal systems).^16^ In a study by Lee et al,^17^ the enrolled patients were divided into a mortality group and a survival group. The results showed that the SOFA score in the mortality group was significantly higher than that in the survival group, suggesting that the SOFA score can predict the prognosis of trauma patients; similarly, another study showed that among 1,554 trauma patients,^18^ the SOFA scores at 24 hours, 48 h, and 72 h were higher in the death group than in the survival group. All of the above studies were similar to the results of the present study; therefore, the SOFA score can be used as an independent risk factor for patient prognosis and has a good predictive value for patient prognosis. The sensitivity and specificity of the SOFA score were found to be superior to the ISS in this study, which is rarely mentioned in previous research. This undoubtedly provides clinicians with an early and easily obtainable effective indicator.

Early uncontrolled bleeding is the leading cause of death in patients with severe unstable pelvic fractures.^19^-^21^ This is also confirmed by the results of this study. The sources of bleeding are venous injury, arterial injury, or bleeding from the fracture end, and the bleeding control strategy should be patient-specific. Depending on the injury and source of bleeding, hemostatic measures alone or in combination may be used to treat and stabilize the bleeding.

The relevant results of this study demonstrated that patient shock index, ISS, SOFA score, and bleeding are independent risk factors for patient mortality. They can provide treatment reference to reduce the mortality rate of patients with hemodynamically unstable pelvic fractures in clinical practice. For such patients, not only should attention be paid to organ function but also to controlling bleeding. Early intervention therapy should be performed for patients with confirmed pelvic fractures accompanied by hemorrhagic shock, and multi-department cooperation is needed to save lives and improve patient prognosis.

### Shortcomings and Limitations:

This study is a single-center study with a limited sample size, and the included clinical indicators are not comprehensive. Future multicenter and large sample studies with more relevant clinical indicators are needed to further investigate the factors influencing mortality in patients with hemodynamically unstable pelvic fractures.

## CONCLUSION

In conclusion, patients with hemodynamically unstable pelvic fractures are predominantly young and middle-aged males and mortality is high. Shock index, ISS, SOFA score and bleeding are independent risk factors for patient death. A complete understanding of the epidemiologic characteristics of these patients is important for early identification of high-risk patients and reduction of patient mortality.

### Author’s Contribution:

**DZW:** Study design, data collection and analysis. Literature search.

**DZW, JGX & KFZ:** Critical review, Manuscript preparation, drafting and revising.

**DZW & KFZ:** Literature search, Critical Review and final approval of manuscript.

**DZW:** Responsible and accountable for the accuracy or integrity of the work.
